# Transactions of the New York State Society

**Published:** 1895-12

**Authors:** 


### Transactions of the Homœopathic Medical Society of the State of New York for the year 1894. Volume XXIX. Edited by the Secretary, John L. Moffat, M. D., Brooklyn, N. Y.

This volume is, as stated on the title-page, the twenty-ninth annual report of this well-known and influential society. It makes a volume of over four hundred pages, full of interesting and valuable papers, the number of which is too great to catalogue in this notice, much less to discuss. Copies may be obtained of the Secretary, Dr. John L. Moffat, 17 Schermerhorn St., Brooklyn, N. Y.

